# A Novel Missense Mutation 224G>T (R75M) in *SRY* Coding Region Interferes with Nuclear Import and Results in 46, XY Complete Gonadal Dysgenesis

**DOI:** 10.1371/journal.pone.0168484

**Published:** 2016-12-28

**Authors:** Wufang Fan, Bei Wang, Shanshan He, Tengfei Zhang, Chenxing Yin, Yunping Chen, Shuqi Zheng, Jixia Zhang, Lin Li

**Affiliations:** 1 Hebei University School of Life Sciences, Baoding, Hebei, China; 2 Institute of Medical Genetics, Linyi People’s Hospital, Shandong, China; German Cancer Research Center (DKFZ), GERMANY

## Abstract

*SRY*-mutation-caused sex reversal is a rare disease and mostly associated with a *de novo* mutation since the patients with defective *SRY* is infertile. There are many reports about *SRY*-mutation associated 46, XY ovarian disorder of sex development (DSD), but few described their molecular mechanism. Here we report a *de novo* mutation 224G>T (R75M) in *SRY* associated with a phenotypic female, 46, XY karyotype and dysgerminoma. The wild and mutated *SRY* were cloned into recombinant plasmid and expressed in cells *in vitro*, the result showed the mutated SRY is greatly accumulated in cytoplasm while the wild type SRY is mostly localized in nucleus. To make sure no other genes were involved, we performed the trio-based whole exome sequencing using the DNA samples from the proband and the parents, and no mutations were identified especially in *DHH*, *NR0B1*, *NR5A1*, *SOX9* and *MAP3K1*, indicating the *de novo* mutation in *SRY* is the single defect responsible for the female sex reversal. We also used bioinformatics simulation analysis to predict impact of the mutation on *SRY* function, and find the R75 in wild type SRY can form a hydrogen bond with serine at 91 (S91) that make the SRY protein well fit into the minor groove of target DNA, while the M75 in the mutated SRY can’t. Finally, we reviewed *SRY* mutations based on the available references and analyzed the mutation distribution patterns according to density and continuity, which may be useful for further study of the SRY structure, function, and its relatedness with DSD.

## Introduction

XY gonadal dysgenesis is also known as Swyer syndrome. The patients show a 46 XY karyotype but express a female phenotype externally. The patients with the syndrome also have a high risk of tumors such as dysgerminoma and gonadoblastoma in the gonads [1, 2]. It is believed that about 10–15% of the disease is caused by SRY mutation [3].

*SRY* is an essential gene in male mammals to initiate male sexual differentiation and determine testis development [4, 5]. It locates on the end of short arm of the Y chromosome and encodes a protein of 204 amino acids. Interestingly, it is not a typical eukaryotic transcript unit but a single exon-containing gene without intron. The SRY protein contains a single homeobox domain called high mobility group (HMG), which is the most important part in SRY, and majority of the reported mutations occur within it. SRY plays a role as transcription factor [6]. It is assumed that SRY works as a DNA binding factor affecting local chromatin structure approximate to its target genes to boost transcription initiation [7, 8]. In XY fetus, a defective *SRY* fails to activate its target genes, in turn the indifferent gonad can’t differentiate into testes. Without testes, the XY fetus will develop into a baby with male-to-female sex reversal and high risk of bilateral or unilateral dysgerminoma and gonadoblastoma.

*SRY* is one of the genes with intensive mutations in human genome: a gene encodes 204 amino acids while 80 mutations were reported so far, and most of the mutations cause a defective phenotype, i.e., complete or partial SRY-related 46, XY gonadal dysgenesis. Even with so many mutations were reported, very little is understood about how the mutations affect SRY function at the molecular level.

We report a novel missense mutation identified from a patient with 46, XY complete gonadal dysgenesis. The mutation, 224G>T, locates in the N-terminus of HMG domain which changes the amino acid at position 75 from arginine to methionine (R75M). We performed whole genome trio-exome sequencing to exclude other genes involved, and then conducted GFP (green fluorescent protein)-SRY fused protein expression to observe its nuclear import function in transfected cells. We also conducted bioinformatics simulation to analyze how the mutation impacts on SRY-DNA interaction. Finally we reviewed the available references about SRY mutation. Here is our report.

## Case Report

The patient is a 20-years old woman. Her main complaints are primary amenorrhea and abdominal masses. Physical examination showed 156cm in height and 56kg in weight, less subcutaneous fat than a typical woman, hypoplastic breast, and external female genitalia with public hair in inverted triangle. No touchable masses were found in bilateral canalis inguinalis and labium majus. The abdomen was soft and a smooth-surfaced mass was touched one each side which doesn’t show any tenderness or rebound tenderness but a shifting dullness when percussed. A surgery of bilateral ovarian tumor hysterectomy was performed for the patient. The tumors was 10cm×6cm×5cm on right side and 25cm×22cm×10cm on left side, both were attached with streak gonad and normal fallopian tube. Histological examination revealed the tumors belong to dysgerminoma.

## Laboratory Examination

Serological test was done. Human choriogonadotropin (HCG) was 14.5 mIU/mL (normal range for female <3.0 mIU/mL); prolactin (PRL) was 3.2 nmol/L (normal range for female 0.08~1.00 nmol/L); follicle-stimulating hormone (FSH) was 35.0 U/L(normal range for female 2.8~14.4U/L); luteinizing hormone (LH) was 23.4U/L (normal range for female 1.1–11.6IU/L); and testosterone (T) was 7.3 nmol/L (normal range for female 0.97–38.41nmol/L). The Caner Antigen-125(CA125) was 278KU/L (the normal range<35 KU/L).

## The Ethics Consideration

This study was performed at Hebei University and Linyi people’s hospital in Shandong Province. The institutional ethical review committees (Ethics Committees of Hebei University and Linyi People’s Hospital) approved the study protocol, and the proband and her relatives provided written informed consent.

## Materials and Methods

### Cytogenetic analysis

Lymphocytes from peripheral blood samples were cultured for 72 hours in RPMI-1640 medium supplemented with 10% fetal bovine serum. G-banding was performed with Olympus BX53 microscope (Olympus, Japan) equipped with Beion karyotype analysis software (Beion Medical Technology, Shanghai, China) following the routine procedure. At least 50 metaphase cells were analyzed, and 5 metaphases were photographed to determine the patients' karyotype that was named according to ISCN [9].

### DNA preparation and sequencing

*SRY* is a single-exon-containing gene without any intron. To perform a DNA sequencing analysis of *SRY* gene, genomic DNA was extracted from the peripheral blood of patients and controls using standard procedure. To detect the *SRY* presence, one pair of primers was designed that consists of forward primer 5’-GCAGT AGAGCAGTCAGGGAG-3’ beginning from the -130bp upstream of *SRY* exon, and reverse primer 5’-GGCAGGCTCACTTCTGGATG-3’ containing 96bp of genomic DNA downstream of *SRY* exon. The primers amplified a PCR product with 1113bp in full length. For DNA sequencing, two pairs of *SRY* primers were designed. The first pair consists of the forward primer 5’-GCAGTAGAGCAGTCAGGGAG-3’ from +130 to +111bp upstream the exon, and the reverse primer 5’-GGATCTGCGG GAAGCAAAC-3’from 584 to 566bp of the exon. The second pair consists of the forward primer 5’-GCTCTTCCTTCCTTTGCACTG-3’ from 230 to 250bp of the exon, and the reverse primer 5’-GGCAGGCTCACTTCTGGATG-3’ from 96 to 77bp of flanking genomic DNA downstream of the exon. The predicted PCR product is 714bp for the first pair of primers and 754bp for the second pair respectively, and the DNA products from the two pairs of primers are overlapped with 355bp in length. The PCR was performed and then the products were sent for sequencing by Sanger method in both directions (BGI, Shenzhen, China). The sequencing result was analyzed and compared with human *SRY* gene (RefSeq NM_003140.2, NCBI) by BioEdit Sequence Alignment Editor 7.0.5.3 [10].

### Trio-Whole-Exome sequencing

Adult members of the study family, the proband, mother, and father, provided written informed consent for themselves in the study. The study protocol was approved by the Hebei University Medicine Institutional Review Board in accordance with the Declaration of Helsinki. The paired-end whole-exome-enriched libraries were prepared from genomic DNA isolated from the peripheral blood of the proband and parents using BloodGen Mini Kit (Cat# CW2087, Kang Wei, Beijing) and were sequenced on the Illumina Genome Analyzer IIx (Illumina, San Diego, CA), and sequencing reads (with 20× or more coverage in 95% of targeted regions) were aligned to the June 28, 2013 human reference assembly (GRCh37.p13). Annotated high quality variants were subsequently filtered to exclude common variants (>1% minor allele frequency). Variants of interest were subsequently confirmed by di-deoxy sequencing.

### Recombinant plasmid construction, cell culture and transfection

Mutant and wild type *SRY* coding region was cloned into pZsGreen1-N1 plasmid as recombinant plasmid pZsGreen1-N1-*SRY* and pZsGreen1-N1-R75M respectively. The *SRY* coding region DNA was created by PCR with forward primer 5’- CGGAATTCATGCAATCATATGCTTCTGC-3’ containing a *Eco*RI restriction site and an ATG codon, and reverse primer 5’-CGGGATCCCGCAGCTTTGTCCAGTGGC-3’containing a *Bam*HI restriction site without TAG site in order to fuse with GFP. The PCR product was inserted into the pZsGreen1-N1 vector at *Eco*RI and *Bam*HI sites. All constructs were verified by sequencing.

Human gastric adenocarcinoma cell line MGC803 was purchased from the Institute of Basic Medical Sciences under Chinese Academy of Medical Sciences (Beijing, China). The cell line was maintained in high glucose DMEM with 10% fetal bovine serum (FBS), 100IU/ml penicillin G and 100μg/ml streptomycin at 37°C in a humidified incubator with 5% CO2.

MGC803 cells seeded in twelve-well plates with round coverslip were transfected with 1μg per well of pZsGreen1-N1 plasmid, the pZsGreen1-N1-*SRY* (wild type) and the pZsGreen1-N1-R75M (mutant) using Lipofectamine 3000 Reagent (Invitrogen, USA). After 24h, cells were washed three times with PBS, and then fixed in 4% paraformaldehyde (Solarbio, Beijing) for 30 min at room temperature. After three 5-min washes with PBS, the cells were incubated with 0.1 μg/ml DAPI (Sigma, USA) at room temperature for 10 min. The cells were then washed three final times with PBS for 5 min/each wash and observed using a laser scanning confocal microscope (Olympus FluoView, FV1000, Japan).

### Bioinformatics simulation

The fast dreiding force field was used to simulate the wild SRY and mutant SRY with slight modification and optimization [11]. The simulation analysis was based on pdbID 1G46 that was the result of NMR structural analysis on SRY (57-140aa) bound to target DNA.

## Results

### The female patient has a XY karyotype

**Fig 1:**
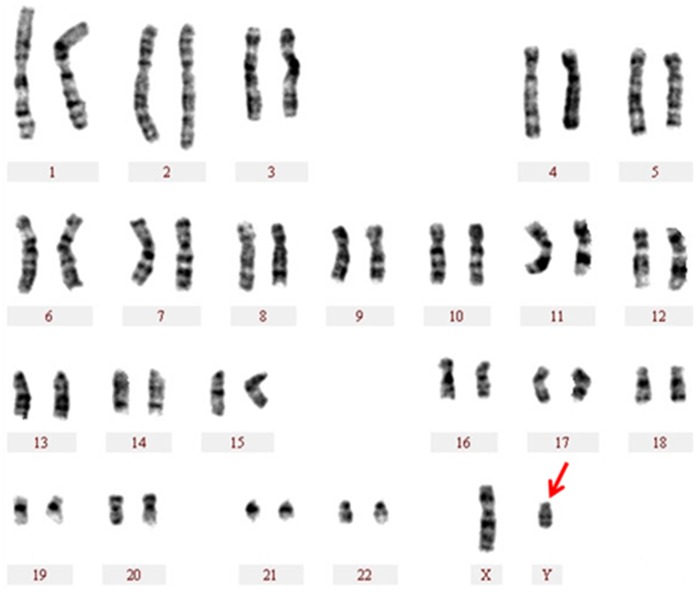
Karyotype of the proband. The Y chromosome is marked by a red arrow.

To understand the possible genetic cause of the defect, a G-banding analysis was first performed on peripheral white blood cells from the patient that revealed a 46, XY karyotype (Fig 1).

### PCR detection of SRY gene

**Fig 2:**
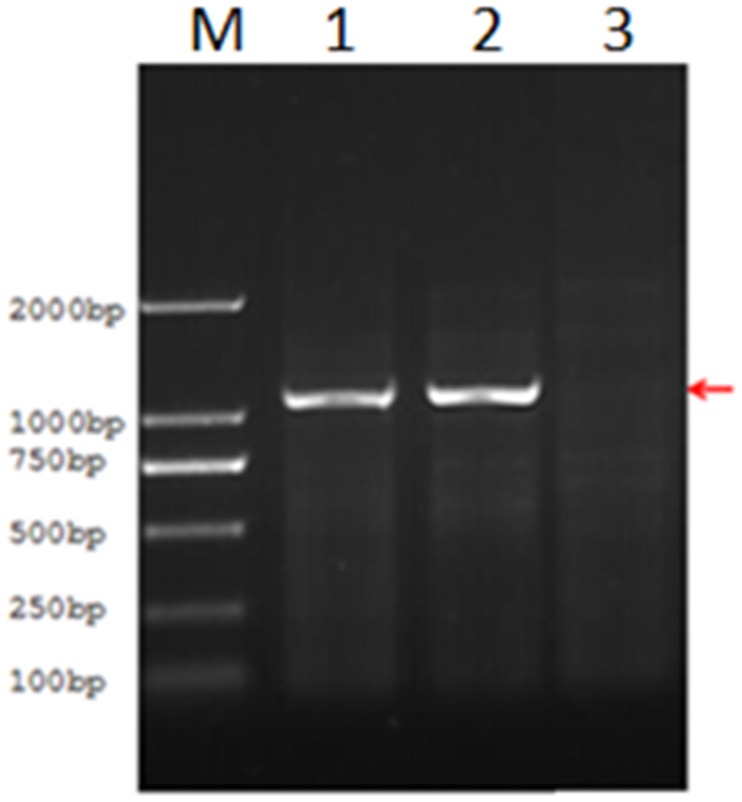
Gel Electrophoresis of PCR Product with SRY-Specific Primers. Lane-1 represents the normal male; Lane-2 the proband; and Lane-3 the negative control (everything except template DNA).

To exclude the possibility that *SRY* gene be deleted in the patient Y-chromosome, we designed *SRY*-specific primers and conducted PCR amplification. The result showed a full-length *SRY* gene does exist in Y chromosome (Fig 2). Based on this fact, the 46, XY complete gonadal dysgenesis was diagnosed.

### DNA sequencing revealed SRY point mutation

**Fig 3:**
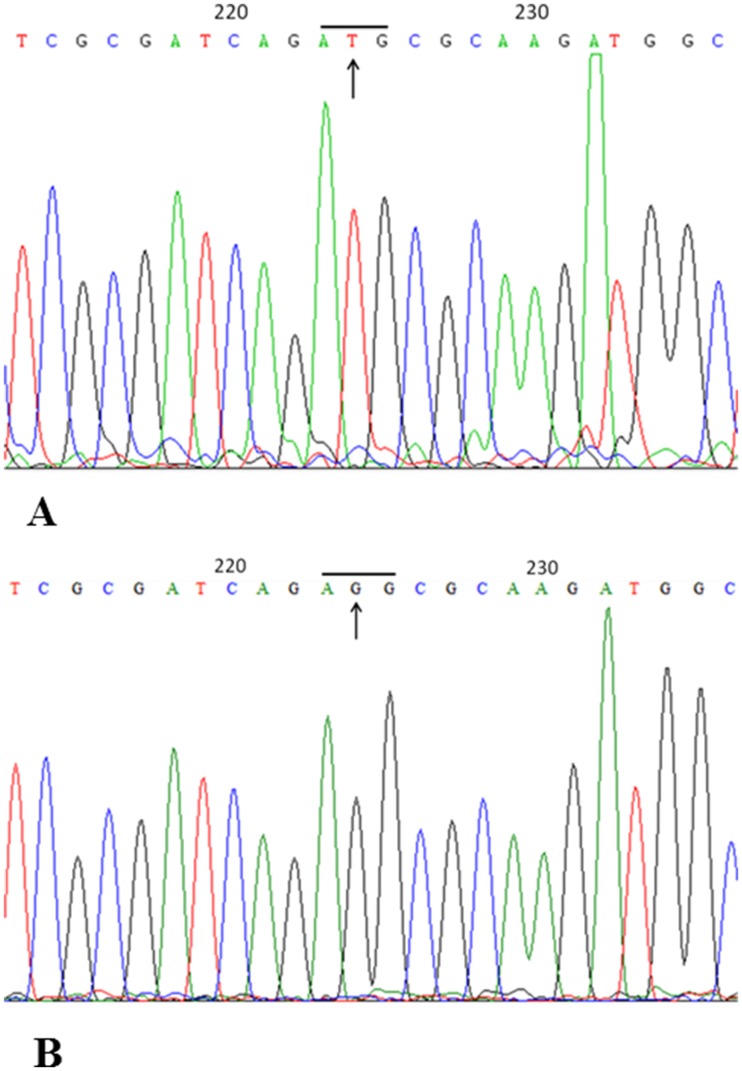
DNA Sequencing to identify Point Mutation. **A**. The mutation 224G/T is identified in proband's SRY; **B**. No sequence change is identified in proband’s father.

**Fig 4:**
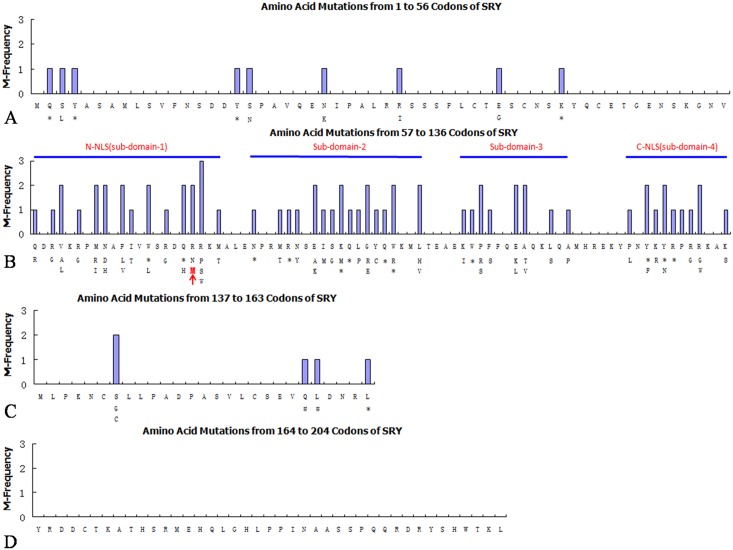
The Point Mutations Distribution in SRY. **A**. Mutations occurred in SRY from 1^st^ to 56^th^ amino acids (AAs) upstream the HMG domain. A total of 9 mutations were out of 56 AAs (16.07%) including 5 missense mutations (5/9, 55.6%) and 4 nonsense mutations (4/9, 44.4%); **B**. Mutations occurred in SRY from 57 to 136 AAs covering the whole HMG domain. A total of 66 mutations were out of 80 AAs (82.5%), including 54 missense mutations (54/66, 81.8%), 12 nonsense mutations (12/66, 18.2%); the novel mutation described in this paper is highlighted by red color and arrow; **C**. The mutations occurred in SRY downstream of 3’ HMG box from 137 to 163 AAs. A total of 5 mutations were out of 27AAs (18.5%); including 2 missense mutations (2/5, 40%), 1 nonsense mutation (1/5, 20%), and 2 frame-shift mutations (2/5, 40%). **D**. No mutations were reported in SRY from 164 to 204 AAs. **Note:** the first row of AAs in each figure represents amino acid sequences of the normal SRY protein, the AAs beneath the first row represent the mutated amino acids; the pound sign# represents frame shift, and asterisk* represents nonsense mutations. Y-axis indicates mutation frequencies, and X-axis marks AAs sequence in SRY protein.

To verify if the *SRY* gene contains mutations, we sequenced the full gene after PCR amplification. To confirm the sequencing accuracy, DNA samples were sequenced three times from different batches of PCR product. The results were consistently showed the same point mutation in the proband but not in the father (Fig 3). A missense mutation, 224G>T, was found in *SRY* coding region of the proband, and resulted in the arginine codon AGG substituted by methionine codon ATG at amino acid (AA) position 75, or R75M in SRY protein. The available data about mutations reported by others that caused 46, XY complete or partial gonadal dysgenesis were summarized in Fig 4.

### Whole-Exome Sequencing (WES)

To explore the possibility that the defect-causing mutations occur in genes other than SRY, we performed the trio-based whole exome next-generation sequencing. The detailed analysis didn’t identify any variants in other genes that are possibly associated with 46, XY complete gonadal dysgenesis, especially in *DHH*, *NR0B1*, *NR5A1*, *SOX9 and MAP3K1*. This confirmed the *SRY* 224G>T (R75M) is the only sequence variant involved in sex reversal.

### Impact of mutation 224G/T (R75M) on SRY Nuclear Localization Signal (NLS)

**Fig 5:**
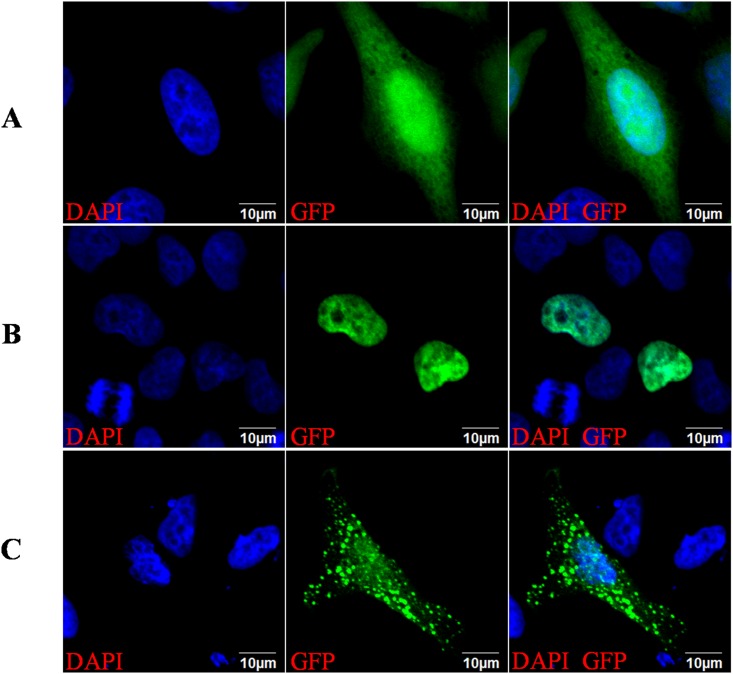
Impact of R75M Mutation on SRY Nuclear Import. MGC-803 cells were transiently transfected to express GFP and GFP-SRY fusion proteins. Photos were taken 24h later by laser scanning confocal microscope (40 x magnifications). **A**. Laser confocal microscope observation of pZsGreen1-N1, showing the GFP is distributed mainly in cytoplasm; **B**. Laser confocal microscope observation of pZsGreen1-N1-*SRY*, showing the overwhelming accumulation of WT-SRY in nucleus; **C**. Laser confocal microscope observation of pZsGreen1-N1-R75M, showing both nuclear and cytoplasm accumulation of mutant SRY protein as granules or clumpy spots.

To understand more about impact of the novel mutation R75M on SRY function, we conducted experiment to test if the mutant SRY has any influence on SRY translocations in cell. The ORFs of wild type *SRY* and mutant *SRY* (R75M) were cloned into a GFP-tagged plasmid pZsGreen1-N1 separately; then the MGC-803 cell line was transfected with the recombinant plasmids respectively; finally the GFP-tagged SRY were observed under laser scanning confocal microscope. The results showed that the mutant SRY localize more in cytoplasm than in nucleus while the wild type SRY accumulates only in nucleus. It indicates that R75M mutation has a significant inhibition of nuclear import, and the arginine at position 75 of SRY is associated with NLS [12] function (Fig 5). From the microscope observation, we also find that the mutant protein appeared as granules or clumpy spots in both cytoplasm and nucleus instead of an even distribution as the normal SRY protein does, which suggests that the R75M mutation may decrease solubility or hydrophilicity for SRY protein.

### 224G/T (R75M) mutation impairs SRY-DNA binding capability predicted by bioinformatics simulation analysis

**Fig 6:**
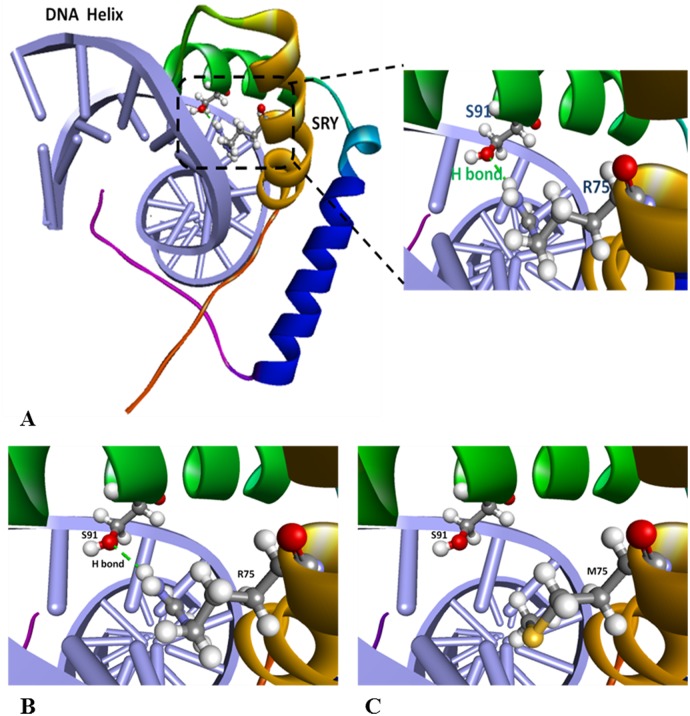
Simulation Analysis of R75M Point Mutation that Causes Impaired DNA-Binding Capability. **A**. The arginine at position 75 (R75) can form a hydrogen bond with serine at position 91 (S91) which fits well with the minor grove of target DNA. **B**. Showing how the R75 form a hydrogen bond with S91 in detail. **C**. When arginine (R75) is substituted by methionine (M75), the hydrogen bond with S91 is destroyed.

To understand more about the impact of novel mutation 224G>T (R75M) on SRY-DNA interaction, we used bioinformatics simulation analysis to predict conformational change caused by the mutation. The result shows that the R75 in wild type SRY can form a hydrogen bond with 91S (serine at position 91 of SRY) that cooperatively maintains a perfect conformation fitting into the minor groove of target DNA helix, while after the 75R (arginine) mutated into 75M (methionine), the latter can’t form a hydrogen bond with 91S, which destroyed the obligated protein conformation, and the capability of SRY-DNA binding was aborted (Fig 6).

## Discussion

We have reported a novel mutation in the *SRY* gene from an XY female with complete gonadal dysgenesis. This mutation takes place at nucleotide position 224 of the SRY coding region, where a guanine in the second position of the codon is replaced with a thymine (224G>T). At protein level, this mutation substitutes an arginine with a methionine in amino-acid position 75 of the SRY protein (R75M). Genomic DNA sequencing shows the variant is not a familial trait, and trio-based whole exome sequencing confirmed the SRY mutation 224G>T is the only genetic defect for the patient. Normal male sex determination in mammals is mediated by functional *SRY*, and a defective *SRY* can cause failure of testicular development that, in turn, results in gonadal dysgenesis and sex reversal phenotype.

The mutation 224G>T (R75M) locates at N-terminal of the HMG box, where exists a functional NLS [13, 14, 15, 16, 17, 6, 18]. Our experiment showed the mutant protein was severely inhibited for nuclear import and greatly accumulated in cytoplasm. This is the first report about R75M mutation can block functional NLS with experimental evidence. In additions, codon 75 locates at the center of a cluster of 3 mutations (Fig 4B), suggesting it may play a vital role in NLS. Beside nuclear import, we also find that the R75M mutation can diminish SRY-DNA binding because mutant R75M can’t form a hydrogen bond with serine at position 91 so that the SRY conformation necessary for SRY fitting into DNA minor groove was aborted. We believe that the NLS and DNA-binding defects both contribute to sex reversal. Firstly, only partial SRY protein can access to nucleus which affects their efficiency to promote assembly of transcriptional complex; secondly, the DNA-binding defect adds up and causes an additive effect which doubly ensures the interruption of sex differentiation cascade. This is the first report to explain the molecular mechanism about how the R75M mutation can cause sex reversal through diminishing both NLS and DNA-binding capabilities of SRY in a molecular conformation level.

For a transcription factor to exert its regulatory role, it must move and localize in the nucleus after it is synthesized in cytoplasm. This process is called nuclear import. Nuclear import is mediated by NLS motif which consists of short stretches of basic amino acids. In SRY protein, R75 stands side by side with R76 and K77 to form a trio basic amino acids RRK, which exactly satisfy the NSL sequence requirement [19]. It is believed that the DNA binding domains in HMG-box consists of three alpha helices separated by loops [20]. Based on our prediction, R75 is one of the amino acids that locates at middle of the first alpha helix at N’-terminus and form a hydrogen bond with S91 from the second alpha helix which fits into the minor groove of the target DNA as a finger-like protrusion (Fig 5). Our prediction meets the criteria for DNA binding domain in HMG perfectly. It is possible that NLS and DNA-binding motif may be mutual inclusive in SRY.

It has been known that the SRY-specific target DNA sequence is AACAAAG [21] or [T/A]AACAA[T/A] [22] that are accommodated in a linear DNA duplex as B-type DNA. After SRY-DNA binding, the target DNA will produce a sharp bend with widened minor groove which facilitate alpha-helixes from SRY HMG box intercalating into it. The DNA bend will be recognized by transcriptional factors and a transcriptional complex will be assembled [23, 24]. R75M mutation distorts the natural conformation of the SRY alpha-helixes, so that it can’t insert into minor groove of the target DNA to maintain a sharp DNA bend. As a result, the mutated SRY will lose its key function in the male sex differentiation cascade. Our findings are consistent with those references very well.

To see if other genes involved in the sex reversal as we reported, we performed trio-based whole exome sequencing, and the result excluded such possibility. It suggests 224G>T (R75M) mutation is the only genetic error to cause the described defect. Clearly, *SRY*-associated gonadal dysgenesis is one of the Mendelian disorders with high penetrace because majority of the *SRY* mutations can cause a defective and self-evident phenotype and only a few *SRY* mutations were reported not affecting sex development. It is estimated that human genomes typically contain ~100 genuine LoF (Loss of Function) variants with ~20 genes completely inactivated and approximately 5 common complete knockout genes in autosomes which may be associated with disorders or harmless [25, 26, 27]. However, we don’t know how many complete knockouts happening in sex chromosomes, especially in Y-chromosome. *SRY* mutation may be one of the human knockouts occurred in Y chromosome.

Human *SRY* is a simple gene package without any intron. It consists of three parts, i.e., N-terminus part (1-56codons), HMG domain (57–136, 80codons), and C-terminus part (137–204, 68codons). We collected and reviewed all available data on *SRY* mutation until recently [28]. A total of 80 mutations were reported in 204AAs of the full length SRY, which show 0.3921 mutation per codon at average (80/204). The mutations are not occurring as a random fashion. The 80 mutations occurred in 59 codons because of triple mutation in 1 codon (1.7%, 1/59), double mutations in 19 codons (32.2%, 19/59), and single mutation in 39 codons (66.1%, 39/59), which indicate some of codons mutate more often than others. We believe the more often mutated codons may carry more vital function in *SRY*. Among the 80 mutations reported, 66 mutations are concentrated in the HMG domain, which takes 82.5% (66/80) of the total mutations (Fig 4). The 66 mutations within the HMG domain actually occur in 46 codons, including 54 missense mutations (81.82%, 54/66), 12 nonsense mutations (18.18%, 12/66), 18 double mutations (39.13%, 18/46) and 1 triple mutation (2.1%, 1/46). Based on the continuity and density of mutations, the HMG can be divided into 4 sub-regions separated by 3 or more consecutive non-mutated codons (Fig 4B). The first sub-region covers 22 codons (from 57 to 78) that include 14 codons carrying 23 mutations due to 1 triple and 7 double mutations and 7 codons as mutation-free (Sub-domain-1 in Fig 4B). A cluster of 3 codons from 74 to 76 show consecutive mutations without interruption. The *de novo* mutation R75M occurs at middle of this cluster. The second sub-region spans 20 codons (from 82 to 101). Among them, 14 mutations were reported in a cluster of 10 consecutive codons from 87 to 96 due to 4 double mutations (Sub-domain-2 in Fig 4), indicating all these amino acids exert an important function in a synergic way so that change of any one will result in a loss of function. The third sub-region covers 13 codons (107 to 119) with 11 mutations. Among them, 5 mutations were reported in a cluster of 4 continuous codons (107–110) due to one double mutation (Sub-domian-3 in Fig 4B). The fourth sub-region covers 12 codons (125 to 136) with 12 mutations. Among them, 10 mutations were located in a block of 7 consecutive codons (127 to 133) due to 3 double mutations (Sub-domian-4 in Fig 4B). Based on the mutation distribution patterns, we hypothesized that the N-terminus NLS may covers 22 amino acids from 57 to 78, and the C-terminus NLS may cover 12 codons from 125 to 136. The epitopes that determine the SRY-DNA interaction may be strongly associated with 10 codons from 87 to 96, and 4 codons from 107 to 110 respectively. This description is entirely inferential; and the actual identity is best determined experimentally. However, it is consistent with our prediction that the codon 91 encoding S91 to form a hydrogen bond with R75 exactly locates at center of subdomian-3 from codons 87 to 96.

Interestingly only 9 mutations were reported upstream the HMG domain in the first 56 codons of *SRY*, which include 3 nonsense mutations and 6 missense mutations, and all of the mutations are occurred in the first 43 codons (Fig 4A). The nearest nonsense mutations were reported at 2nd and 4th codons, which are understandable; but the puzzle is that the nearest missense mutation occurs at 3rd codon and causes a XY, complete gonadal dysgenesis too [29].

Only 5 mutations were reported immediately downstream HMG from codons 137 to 163 including 2 missense mutation at codon 143 due to double mutations, 2 frameshift mutations at codons 158 and 159, and 1 nonsense mutation at codon 163 (Fig 4C). No mutations were reported so far in the last 41 codons from 164 to 204 that takes 20% (41/204) of the full length SRY (Fig 4D). This observation suggests some amino acids proximal downstream HMG still play crucial roles to keep SRY functional; and the amino acids at distal downstream HMG may be less functional. However, the puzzle is that if the last 41 amino acids contribute little to SRY function, why the truncated SRY by nonsense mutation at codon 163 can cause XY complete gonadal dysgenesis as reported? [30]. The same situations can be applied to the frameshift mutation at codons 158 and 159. It is very interesting to know that the missense mutation S143C can diminish SRY-DNA interaction [31], it mean the amino acids outside HMG domain can still influence *SRY* function by certain ways.

In conclusion, we identified a novel point mutation, 224G>T (R75M), in *SRY* coding region that causes a 46, XY complete gonadal dysgenesis with bilateral dysgerminoma. We tested the impact of the mutation on *SRY* function and demonstrated the R75M mutation greatly blocks *SRY* nuclear import. Our bioinformatics analysis shows that the R75M mutation may damage SRY-DNA interaction by impeding hydrogen bond between R75 with S91. This is the first report about mutation R75M in *SRY* and the first to explain how the mutation R75M can block SRY-DNA binding at molecular conformation level. Our review summarized the available SRY mutation data and analyzed their distribution pattern in details, which will be helpful for further tackling of *SRY* function and its diseases-relatedness.
